# First Report of *Leptopilina japonica* in Europe

**DOI:** 10.3390/insects11090611

**Published:** 2020-09-08

**Authors:** Simone Puppato, Alberto Grassi, Federico Pedrazzoli, Antonio De Cristofaro, Claudio Ioriatti

**Affiliations:** 1 Technology Transfer Centre, Fondazione Edmund Mach, Via Edmund Mach 1, 38010 San Michele all’Adige, Trento, Italy; simone.puppato@fmach.it (S.P.); alberto.grassi@fmach.it (A.G.); federico.pedrazzoli@fmach.it (F.P.); 2 Department of Agricultural, Environmental and Food Sciences, University of Molise, Via Francesco De Sanctis, 86100 Campobasso, Italy; decrist@unimol.it

**Keywords:** Figitidae, biological control, larval parasitoid, cherry, *Drosophila suzukii*

## Abstract

**Simple Summary:**

The invasive spotted wing drosophila, *Drosophila suzukii*, is a polyphagous species that has become a serious fruit pest worldwide. Biological control plays a key role in the integrated management of invasive insect pests. With the aim to verify whether some parasitoid wasps, being considered as major mortality factors for *D. suzukii*, followed its host along global pathways, a field survey was conducted by sampling fruits and installing fruit-baited traps near isolated cherry trees within a wide agricultural area. Morphological and molecular analysis revealed three specimens of *Leptopilina* *japonica* in the parasitoid complex collected during the 2019 field survey; this is considered the first record of this larval parasitoid in Europe. A wider survey carried out during 2020 confirmed the presence of an adventive population of the parasitoid. In terms of reproductive biology, *L. japonica* shows similarity with *Ganaspis* *brasiliensis*, the best candidate for a future program of classical biological control. Interplay among indigenous parasitoids and the newly arrived Asian parasitoid, as well as the interactions of these species with *G. brasiliensis*, offer a unique ecological context to acquire new insights into the relationship between *D. suzukii* and its natural enemies and into their role in providing effective control of the pest.

**Abstract:**

*Drosophila suzukii* (Matsumura; Diptera: Drosophilidae) is a key pest of sweet cherry and small fruits worldwide. Biological control remains unutilized in the framework of *D. suzukii* management. Nonetheless, natural enemies may play an important role in regulating this pest. We report for the first time the presence of *Leptopilina japonica* Novković and Kimura (Hymenoptera: Figitidae) in Europe. Two specimens emerged from ripened fruits and one was collected after direct observation on a cherry tree in June 2019. They showed the distinctive morphological traits already described and shared more than 99% sequence similarity with specimens of *L. japonica* collected in Asia. This first finding was confirmed by a wider survey carried out in 2020; *L. japonica* emerged from cherry fruit samples collected in five other sites across the Trentino region, suggesting that *L. japonica* has already colonized a wide area. Detection of this Asian species is relevant to the future direction in managing *D. suzukii*, both in Europe and North America. In fact, *L. japonica* showed similarity with *Ganaspis brasiliensis* (Ihering) (Hymenoptera: Figitidae), the most promising candidate for the classical biological control, in terms of developmental time, egg maturation, host age preference and lifetime fecundity.

## 1. Introduction

*Drosophila suzukii* Matsumura (Diptera: Drosophilidae), also known as the spotted wing drosophila (SWD), belongs to the ever-expanding group of invasive alien species (IAS) [1]. Native to East Asia [2], *D. suzukii* has rapidly spread both in the Americas and in Europe, where it has become a serious pest of stone and soft fruit crops, causing huge annual crop losses [3].

In Italy, it was recorded for the first time on raspberries in the Trentino region in 2009 [4], and it became an economically important pest on several species of cultivated berries of the region, generating up to €3 million in losses in 2011 [5]. Economic costs of *D. suzukii* include crop loss in the field, increased labor, chemical input costs for monitoring and management, cost of the secondary selection of fruits in the storage facilities after harvest, as well as losses due to the shorter shelf life of fruit containing eggs of *D. suzukii* [6]. The peculiar evolution of a serrated ovipositor [7], as well as of specific behavioral traits [8,9,10,11,12], allowed *D. suzukii* to exploit healthy ripening fruits as substrates for egg-laying, creating a new ecological niche, thus reducing interspecific competition with other Drosophila species [13,14,15]. Polyphagia towards a broad range of cultivated and wild hosts [16,17,18], in addition to high reproductive potential [19,20], has increased its invasiveness in the colonized regions. Finally, the lack of an effective suppression action operated by natural enemies in the newly invaded areas makes possible a rapid building up of the pest population since the beginning of the season. Current control methods rely on integrated pest management (IPM) strategies, among which exclusion netting and chemical insecticides remain the first lines of defense [21,22,23,24,25,26,27,28,29,30,31,32,33,34,35,36,37,38]. However, reiterated applications of pesticides due to *D. suzukii* lifecycle and behavior [23,24,25] may bring some negative side effects [26,27,28].

Generalist predators, pathogens and two cosmopolitan pupal parasitoids, *Trichopria drosophilae* Perkins (Hymenoptera: Diapriidae) and *Pachycrepoideus vindemmiae* Rondani (Hymenoptera: Pteromalidae), proved able to use *D. suzukii* as prey/host [29]. However, their generalist behavior raised several doubts about large-scale effectiveness [30], unless conservation practices [31,32] and optimal releasing time [33] are integrated with advanced control strategies still under development [34,35].

Host–parasitoid interaction is a complex phenomenon of co-evolution influenced by multiple ecological and genetic factors, which have become key elements in biological control process [36,37,38]. Elegant studies have investigated the biological basis of the immune response of *D. suzukii*, clarifying the physiological and molecular mechanisms determining failure [38,39,40,41,42] or success [41,43,44] in the development of parasitoid wasps. Field studies in the newly invaded areas by *D. suzukii* completed the picture [30,45,46,47].

An alternative approach might be classical biological control (CBC), defined as the authority regulated introduction of a biological control agent of exotic origin aiming at permanent control of the invasive pest. In past years, faunistic surveys in East Asia revealed an assemblage of parasitoid wasps able to efficiently parasitize and develop on *D. suzukii* [48,49,50,51,52,53]. Among these, larval parasitoids *Asobara japonica* Belokobylskij (Hymenoptera: Braconidae), *Leptopilina japonica* Novković and Kimura and *Ganaspis brasiliensis* (Ihering) (both Hymenoptera: Figitidae) were the most abundant species attacking *D. suzukii*. Therefore, in-depth studies have been carried out in quarantine facilities in order to establish the most promising candidate for CBC. Special attention has been given to the risk assessment towards non-target species, native to the ecosystem where the foreign candidate will be released [54,55,56,57]. Results identified *G. brasiliensis* lineage G1 [58,59] as the most specialized parasitoid and a petition to release it for the biological control of *D. suzukii* in the United States was submitted to the United States Department of Agriculture (USDA) in 2019. However, as is the case for alien crop pests, their natural enemies might also be unintentionally introduced in new areas where they presumably were not present. Parasitoid wasps of the invasive *Halyomorpha halys* (Stål) (Hemiptera: Pentatomidae) are well known examples [60]. Recently, an exotic parasitoid of *D. suzukii* was also found in Mexico [61].

Here, we report the presence of the Asian larval parasitoid, *L. japonica*, in the Trentino region. This parasitoid shows local adaptation to some sympatric Drosophila species and has been demonstrated to be highly virulent to those considered as its major hosts, included *D. suzukii* [62]. On the other hand, due to its ecological fitting, it can form novel associations with some allopatric Drosophila species in novel environments that can have unpredictable consequences on the biological control of Drosophila populations.

## 2. Materials and Methods

Specimens of presumptive parasitoids of *D. suzukii* were collected from April to July 2019 during a field survey in the Adige Valley (Trento, Italy), at 186 m a.s.l., with the aim of investigating the parasitoids’ complex of the pest. Several sampling points (SPs) (Table 1), near isolated cherry trees, were set across an agricultural area of around 20 hectares, where apple orchards and vineyards represent the main crops.

Surveys were carried out weekly by means of four different sampling methods as follows.

(1) Fresh ripening fruits: a total of 50 fruits were collected from isolated cherry trees found in each SP and were checked under stereomicroscope for *D. suzukii* eggs. Infestation level was expressed as percentage of infested fruits on total fruits inspected. Fruits were considered infested when carrying at least one egg. Infested fruits were placed in plastic boxes equipped with a fine mesh netted lid for ventilation and sand and paper towel as substrates to dry up the juice produced.

(2) Fallen fruits: a total of 30 dropped cherries were sampled from the ground of each identified isolated cherry tree. Fruits were placed onto a plant pot saucer containing a layer of sand and left exposed for seven more days under the tree. After this period, new samples of dropped cherries were collected to replace the old ones, which were brought to the laboratory to recover pupae by sifting the sand and carefully dissecting the fruits. Eventually, each pupa was identified as *D. suzukii* or other drosophilid species based on morphological characteristics and singly incubated in plastic vials.

(3) Sentinel traps: each trap consisted of a small plastic bowl baited with a fresh banana slice infested by thirty third-instar larvae of *D. suzukii*. Each bowl was placed inside a white delta trap and hung at around 30 cm from ground level at each SP.

All samples were kept in plastic boxes at room temperature (22–25 °C) in the laboratory and they were daily inspected. Emerged specimens of *Drosophila* spp. and parasitoids were placed in 99% ethyl alcohol for further identification.

(4) Direct visual: observations on the foliage layer were conducted at around 1.5–2.0 m from ground level. Putative parasitoids were collected with an insect aspirator.

In each SP, the population dynamic of *D. suzukii* was monitored by means of a Drosotrap (Biobest, Westerlo, Belgium) filled with 200 mL liquid bait DroskiDrink (75% apple cider vinegar, 25% red wine) mixed with a teaspoon of raw brown sugar and 100 µl Triton X-100 (Sigma-Aldrich, St. Louis, MO, USA). Traps were hung at 1.2–1.5 m from ground level. Trap bait was weekly renewed and content was inspected under stereomicroscope for *D. suzukii* individual counting.

Temperatures were recorded by means of a data logger placed at the center of the sampling area.

Identification of parasitoid wasps was carried out combining morphological and molecular analysis, in order to achieve robust and informative data. Regarding the first approach, Figitidae species were determined using diagnostic morphological features and identification keys [63,64,65,66,67,68,69].

**Figure 1:**
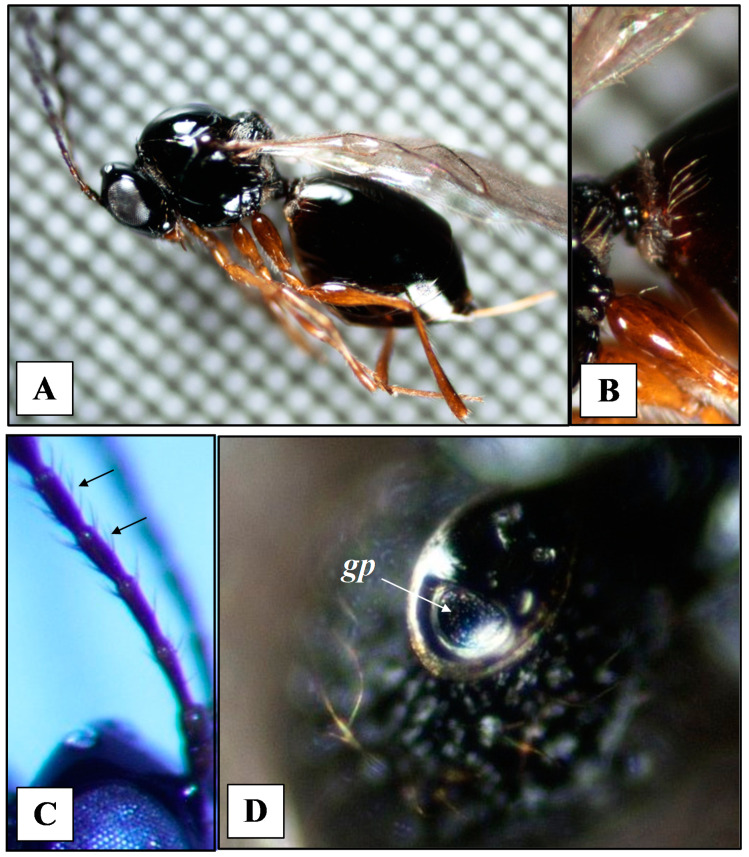
*Leptopilina japonica* female from Trento, Italy: (**A**) Adult habitus, laterally, (**B**) Petiole and metasomal hairy ring, lateral view, (**C**) Female 5th and 6th antennal segments (black arrows) at higher magnification, lateral view (**D**) Scutellar plate, gp: glandular pit.

*Leptopilina japonica* (Figure 1A) can be identified from other common *Leptopilina* species known to occur in Europe by the combination of the following diagnostic characteristics reported in Novkovic et al. [67]: female antennal segments, scutellar plate, posterior pit, metapleural ridges and metasoma with the hairy ring (Figure 1B). In particular, due to the high relatedness of *L. victoriae* and *L. japonica*, antennal segments of *L. japonica* are darker, 5th and 6th antennal segments more slender and longer compared to *L. victoriae* (Figure 1C). Scutellar plate is wider and posterior pit larger than in *L. victoriae*. (Figure 1D).

For the molecular analysis, the total DNA was isolated from each specimen using a commercial kit (NucleoSpin^®^ Tissue; Macherey-Nagel, Düren, Germany). Amplifications were performed with two primer pairs, amplifying the cytochrome oxidase subunit I (COI) gene (LCO1490: 5′-GGTCAACAAATCATAAAGATATTGG-3′; HCO2198: 5′TAAACTTCAGGGTGACCAAAAAATCA-3′) and the internal transcribed spacer 1 (ITS1) (rDNA2:5′-TTGATTACGTCCCTGCCCTTT-3′; rDNA1.58s: 5′-ACGAGCCGAGTGATCCACCG-3′). Polymerase chain reaction (PCR) conditions were as reported in PM 7/76 [70] for COI and in Taylor et al. [71] for ITS1. PCR products, after purification with illustra ExoProStar1-Step (GE Healthcare, Little Chalfont, UK), were sequenced with the Big Dye Terminator v3.1 cycle sequencing kit (Applied Biosystems, Foster City, CA, USA) on an 3130 xl Genetic Analyzer (Applied Biosystems; Carlsbad, CA, USA). A Basic Local Alignment Search Tool (BLAST) comparison of the sequences obtained was performed using the NCBI database to confirm the morphological classification.

In 2020, the survey for parasitoids was extended to five additional sites in the Trentino region. Based on the outcome of 2019, the sampling method was restricted to fresh ripening cherry fruits. A total of 1750 fruits (50 fruits/site × week) were sampled during the weekly survey between May and June (7 weeks) and checked in the laboratory following the procedure of the previous year.

## 3. Results

During the field surveys, a total number of 144 adult parasitoids of different species were collected (Table 2): 53 *P. vindemmiae*, 64 *T. drosophilae* and one *Spalangia* spp. emerged from the sentinel traps, 18 *P. vindemmiae*, 3 *T. drosophilae* and 2 *Leptopilina boulardi* (Barbotin, Carton and Kelner-Pillault) emerged from fallen fruits.

Three voucher specimens, one female and two males of Figitidae, were recovered from ripened fruits (two) and by direct observation (one) on a cherry tree (SP 5) in June 2019. All of them showed the distinctive morphological traits already described by Novković et al. [67] for *L. japonica* (Figure 1) and shared more than 99% sequence similarity with specimens of *L. japonica* collected in Asia [67].

**Figure 2:**
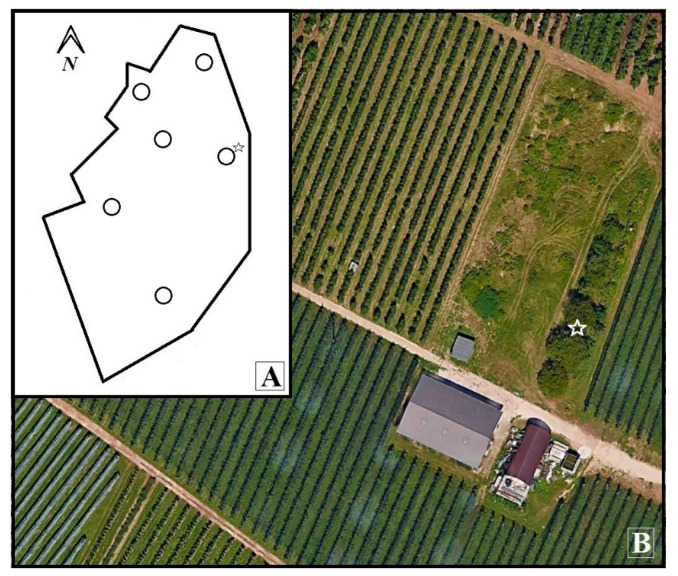
(**A**) Survey area map with sampling points (circles). (**B**) Recovery site of *L. japonica* (star).

The BLAST search on the NCBI database showed the high sequence similarity of our COI barcode sequence with *Leptopilina* sp. *JP* COI pseudogene (accession number: AB546877-78). The ITS1 barcode sequence shared the highest similarity score with *Leptopilina* sp. *JP* gene for internal transcribed spacer 1 (accession numbers: AB546881 and AB583629-31). All specimens showed identical sequences for both molecular markers and a representative sequence was submitted to GenBank (MT840347 and MT840665, respectively). The cherry trees where the specimens were collected were located in the meadow of a farm surrounded by apple orchards (lat. 46.006079° N; long. 11.120367° E) (Figure 2).

**Figure 3:**
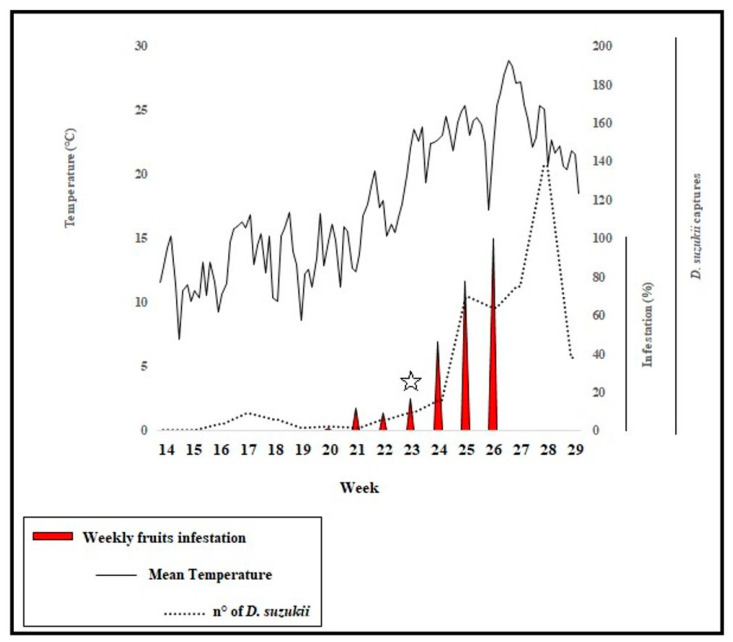
Summary graph of temperature, weekly fruit infestation (% of infested fruits) and *D. suzukii* captures during field survey (flies/trap). The star indicates the week of the *L. japonica* find.

At the time of *L. japonica* findings, the infestation level was around 20% and the mean temperature was around 22 °C (Figure 3).

Results of the 2020 survey (Table 3) confirmed that *L. japonica* is widely established in the region in sites up to around 20 km apart and from 211 to 685 m altitude. A total of 131 parasitoids emerged by the sampled cherry fruits; all of them belonged to the species *Leptopilina japonica.*

## 4. Discussion

*Leptopilina japonica* is a solitary koinobiont endoparasitoid, consisting of two subspecies, *L. japonica japonica* Novković and Kimura present in Northern and Central Japan and *L. japonica formosana* Novković and Kimura occurring in Taiwan [67]. These subspecies seem to have distinct characteristics in terms of behavioral ecology [62,67,72]. Based on morphological and molecular identification, our specimens could be associated with *L. j. japonica* subspecies. The similarity of geoclimatic factors between the native range and the Trentino region may support this speculation.

*L. japonica* was frequently found to parasitize larvae of *D. suzukii* in naturally infested fruits [51,52,67,73]. Field surveys in its native areas also reported that this larval parasitoid attacked *D. suzukii* on different wild and commercial fruits, such as *Prunus* spp., *Myrica rubra, Solanum nigrum, Rubus* spp., *Lonicera maacki, Vaccinium spp., Fragaria moupinensis* and *Sambucus adnata* [51,52,53]. Specimens of *L. japonica* here reported were collected from an unmanaged cherry tree (*Prunus avium*) with 20% of infested hanging fruits. It is noteworthy that this larval parasitoid is foraging at a low infestation level, when the first generations of *D. suzukii* occur on early fruiting non-crop plants.

Furthermore, the field surveys were carried out in a habitat diversity that ranged from inland forest to hedgerow and wild vegetation surrounding cultivations. These ecological features are key aspects for the success of CBC, especially when the target pest exploits a wide range of plant hosts over the season [16,17,18,49]. Understanding habitat and resources preference will improve the experimental design in biological control programs [74].

Concerning reproductive biology studies, *L. japonica* showed similarity with *G. brasiliensis* in terms of developmental time, egg maturation, host age preference and lifetime fecundity, while it developed faster than *G. brasileinsis* for both males and females [75]. Thermal performance of *L. japonica* revealed successful development at constant temperature from 17.2 to 27.3 °C [76]. This thermal range resembles the typical average temperatures occurring in the valley bottom of the Trentino region from the second half of May to September.

Field surveys and pre-release non-target testing with regard to host specificity revealed how *L. japonica* exhibits a wider host range compared to *G. brasiliensis*, leading to exclusion of the former as a candidate agent for CBC [51,57]. Even though a particular emphasis is now placed on host specificity assessments to avoid the introduction of generalist natural enemies [77], laboratory experiments may be limited in their predictive ability [78], due to the confined experimental set-up [79] or because of the existence of strains with a narrow/wide host range according to the area of origin [56,57]. As for other animal species, parasitoid wasps need to cope with the ecological context in which they are found [62,73,80], which provides alternative resources to sustain natural enemies during the season, facilitating biocontrol of target pests [81]. In this sense, if the wider host range of *L. japonica* is confirmed in the open field, it will out-compete the other Asian parasitoids. On the other hand, also an additive effect between *L. japonica* and *G. brasiliensis* [82], which would become an essential aspect to be deepened for controlling *D. suzukii*, has been shown.

Moreover, interplay among exotic and indigenous parasitoids need further attention, particularly *T. drosophilae*, being the current candidate for augmentative biocontrol. If no detrimental effects are observed, one might assume an integration of CBC in the future performed with *G. brasiliensis*, with conservative biological control of both resident parasitoids and the newly arrived species for boosting the suppression of *D. suzukii*.

Beyond the abovementioned information, future investigations have to be addressed in order to verify whether *L. japonica* is in its early phases of establishment in Trentino or whether it has already expanded in the Trentino region and even outside this area. A survey is still ongoing in 2020 with the aim to evaluate the spreading of this Asian parasitoid: the first results reveal that *L. japonica* has emerged from cherry fruit samples collected in five other sites across the Province of Trento, suggesting the existence of an adventive population of the parasitoid. This new ecological situation offers the unique opportunity to acquire new insights for future biological control tactics against *D. suzukii*.

## 5. Conclusions

Globalization of human trade and travel, along with climate change, are important drivers for the redistribution of the insect species around the world, leading in many cases to new threats for agricultural crops worldwide. There is a growing interest in understanding the pathway by which alien species are introduced into novel environments, but the assessment is difficult due to the lack of comprehensive information.

Similarly, alien arthropod predators and parasites, the majority of which arrived unintentionally through natural extra-range dispersal or as contaminants and stowaways [83], are particularly interesting because of their generally positive impact in controlling crop pests of economic importance. Infrequent are the cases that had negative impacts for their interference with other beneficial insects [84].

Even though *L. japonica* was excluded as a possible candidate for the classical biological control programs due to its potentially wider host range, it remains among the most widespread and effective parasitoids of *D. suzukii* in its native range.

Reproductive biological traits and temperature requirements make possible its rapid dispersal and durable establishment in the Alpine region, with an expected positive outcome on biological control of the spotted wing drosophila.

The coming years will be crucial in order to understand the population dynamics that will evolve between this parasitoid’s assemblage and the invasive species in the new ecological setting.

## Figures and Tables

**Table 1:** Sampling points and habitat location.

Sampling Point (SP)	Habitat Type Description
SP1	*Prunus avium* nearby vegetable garden
SP2	Vineyard
SP3	Backyard cherry tree
SP4	Apple orchard
SP5	*Prunus avium* in a lawn surrounded by apple orchards
SP6	Domestic compost nearby henhouse

**Table 2:** Total number of parasitoids collected at the six sampling points during the field survey.

Sampling Week	Total Parasitoids	Parasitoid Species	Host (Parasitoid Number)	Sampling Method	SP (Parasitoid Number)
17/18	4	*P. vindemmiae*	*D. suzukii* (4)	Sentinel trap	SP2(3), SP5(1)
18/19	3	*P. vindemmiae*	*D. suzukii* (3)	Sentinel trap	SP2(2), SP5(1)
21/22	18	*P. vindemmiae*	*D. suzukii* (10)	Sentinel trap	SP6(10)
		*T. drosophilae*	*D. suzukii* (8)	Sentinel trap	SP4(7), SP6(1)
22/23	30	*P. vindemmiae*	*D. suzukii* (4)	Sentinel trap	SP4(1), SP6(3)
		*T. drosophilae*	*D. suzukii* (25)	Sentinel trap	SP4(10), SP5(15)
		*Spalangia* spp.	*D. suzukii* (1)	Sentinel trap	SP6(1)
23/24	10	*P. vindemmiae*	*D. suzukii* (1)	Sentinel trap	SP6(1)
		*T. drosophilae*	*D. suzukii* (9)	Sentinel trap	SP4(9)
24/25	28	*P. vindemmiae*	*D. suzukii* (21)	Sentinel trap	SP3(13), SP4(1), SP5(7)
		*T. drosophilae*	*D. suzukii* (7)	Sentinel trap	SP4(5), SP5(2)
25/26	16	*P. vindemmiae*	*D. suzukii* (1)	Sentinel trap	SP2 (1)
		*T. drosophilae*	*D. suzukii* (15)	Sentinel trap	SP2 (15)
26/27	9	*P. vindemmiae*	*D. suzukii* (9)	Sentinel trap	SP3(4), SP4(1), SP5(4)
23	2	*L. japonica*	*D. suzukii* (2)	Ripen cherries	SP5(2)
25/26	7	*P. vindemmiae*	Other drosophilids (4)	Fallen cherries	SP5(4)
		*T. drosophilae*	*D. suzukii* (2), Other drosophilids (1)	Fallen cherries	SP3(3)
26/27	10	*L. boulardi*	Other drosophilids (2)	Fallen cherries	SP1(2)
		*P. vindemmiae*	*D. suzukii* (2), Other drosophilids (6)	Fallen cherries	SP1(5), SP3(3)
27/28	4	*P. vindemmiae*	Other drosophilids (4)	Fallen cherries	SP1(3), SP3(1)
28/29	2	*P. vindemmiae*	Other drosophilids (2)	Fallen cherries	SP3(2)
23	1	*L. japonica*	*/*	Direct observation	SP5(1)

**Table 3:** Sites and dates of the cherry fruit samplings from which *L. japonica* emerged in 2020 survey.

Sampling Site	Sampling Date	Altitude (m)	Host Fruit	Number of *Leptopilina japonica* Emerged
Lat. 46.18172° N; Long. 11.12434° E	18 May 2020	*211*	*Prunus avium*	31
Lat. 46.04736° N; Long. 11.11347° E	19 May 2020	*200*	*Prunus avium*	58
Lat. 46.09678° N; Long. 11.11846° E	25 June 2020	*195*	*Prunus avium*	10
Lat. 46.04604° N; Long. 11.22987° E	2 July 2020	*476*	*Prunus avium*	23
Lat. 46.13564° N; Long. 11.14373° E	2 July 2020	*685*	*Prunus avium*	9
